# Image processing in digital pathology: an opportunity to solve inter-batch variability of immunohistochemical staining

**DOI:** 10.1038/srep42964

**Published:** 2017-02-21

**Authors:** Yves-Rémi Van Eycke, Justine Allard, Isabelle Salmon, Olivier Debeir, Christine Decaestecker

**Affiliations:** 1 DIAPath, Center for Microscopy and Molecular Imaging, Université Libre de Bruxelles (ULB), Gosselies, Belgium; 2 Laboratories of Image, Signal processing & Acoustics, Université Libre de Bruxelles (ULB), Brussels, Belgium; 3 Department of Pathology, Erasme Hospital, Université Libre de Bruxelles (ULB), Brussels, Belgium; 4 MIP, Center for Microscopy and Molecular Imaging, Université Libre de Bruxelles (ULB), Gosselies, Belgium

## Abstract

Immunohistochemistry (IHC) is a widely used technique in pathology to evidence protein expression in tissue samples. However, this staining technique is known for presenting inter-batch variations. Whole slide imaging in digital pathology offers a possibility to overcome this problem by means of image normalisation techniques. In the present paper we propose a methodology to objectively evaluate the need of image normalisation and to identify the best way to perform it. This methodology uses tissue microarray (TMA) materials and statistical analyses to evidence the possible variations occurring at colour and intensity levels as well as to evaluate the efficiency of image normalisation methods in correcting them. We applied our methodology to test different methods of image normalisation based on blind colour deconvolution that we adapted for IHC staining. These tests were carried out for different IHC experiments on different tissue types and targeting different proteins with different subcellular localisations. Our methodology enabled us to establish and to validate inter-batch normalization transforms which correct the non-relevant IHC staining variations. The normalised image series were then processed to extract coherent quantitative features characterising the IHC staining patterns.

In histopathology tissue-based biomarkers are useful for diagnostic, prognostic, and therapeutic purposes. Many relate to protein expression, which can be evidenced in tissue samples by using immunohistochemistry (IHC). This specific staining technique is based on antigen-antibody reactions and is applied to paraffin-embedded tissue samples. Quantifying IHC staining patterns has thus become a crucial need in pathology practice. For this task, whole slide imaging and automated image analysis have multiple advantages, such as avoiding the effects of human subjectivity in visual evaluation^1^,^2^.

For decades in clinical pathology diaminobenzidine (DAB) has been widely used as a (brown) chromogen for revealing protein expression by means of IHC together with hematoxylin (HEM) for tissue counterstaining. The more antigen-chromogen is present in a tissue area, the darker the area appears albeit with possible signal saturation and without being able to refer to the Beer-Lambert law for DAB staining. However, several studies demonstrate that standardised protocols for tissue processing, IHC, and image acquisition are able to provide DAB staining measurements related to antigen content^3^,^4^,^5^,^6^.

Even inside a given laboratory with a well-controlled workflow, IHC-stained slides may be subject to significant variations during their processing. If several IHC batches are required to process a large slide series, inter-batch staining variations should be reduced as much as possible to allow valid and quantitative staining characterisation across the complete series. As shown in the present study these non-relevant variations are not always easily identifiable by means of visual examination all the while impacting staining quantification by digital analysis. We therefore developed a methodology to identify these variations and to correct them by means of image normalisation. As detailed below, histological image normalisation has been especially investigated for hematoxylin-eosin (H&E) staining. This staining shows the tissue structures in a consistent and specific way (i.e. cell nuclei in blue/violet and other, eosinophilic, structures in red/pink). These properties enable the use of shape information for helping stain identification (e.g., inside and outside ellipse-shaped objects to identify hematoxylin and eosin pixels, respectively)^7^. In contrast, the protein expression patterns evidenced by IHC vary in terms of location, area and intensity, depending on both the tissue analysed and the protein targeted. This makes identification and correction of IHC inter-batch variations, which are not biologically relevant, more challenging. For this purpose we set up a methodology using tissue microarray (TMA) slides, sliced from the same TMA block, to provide staining references for different IHC batches targeting a given protein. After digitisation, a representative sampling is extracted from each reference to capture colour and intensity characteristics. The analysis of the inter-batch variations enables us to rule on the need for image normalisation and to evaluate the actual impact and efficiency of different image normalisation methods that we adapted for IHC staining.

## Previous work and novel contributions

Numerous histological image normalisation methods were specifically developed for hematoxylin-eosin (H&E) staining^7^,^8^,^9^,^10^,^11^,^12^,^13^. In contrast, only a few studies investigated IHC staining^14^,^15^,^16^. The basis for many methods of image normalisation is colour decomposition, a.k.a. deconvolution, to extract appropriate colour vectors. These vectors are extracted independently from each slide series, or staining batch, and then matched to perform colour matching between the different series or batches. This matching operation constitutes an essential step for image normalisation.

In this study we choose an unsupervised context for blind colour decomposition. In contrast, Khan *et al*.^15^ propose a supervised method based on a pretrained classifier. However, this supervised method when applied to H&E staining yields very similar results to the unsupervised approach proposed by Macenko *et al*.^8^. Unsupervised methods for colour decomposition has the advantage to avoid the need to train data for each stain of interest, usually requiring manually-selected pixels in controlled slides from each batch. They are often based on matrix factorization approaches, such as principal component analysis (PCA), independent component analysis (ICA) or non-negative matrix factorization (NMF). These approaches use different kinds of assumptions translated into different constraints and/or objective functions. So PCA aims to account for as much of the data variability while enforcing orthogonality between their components, ICA assumes that each dye stains the tissue independently from all the other dye, and NMF aims to consider the physical constraint that each dye has a non-negative response^14^. Rabinovitch *et al*.^14^ qualitatively compared these three approaches and show that PCA is not appropriate. However, some years later Macenko *et al*.^8^ show that PCA has the ability to identify a plane appropriate for describing the clouds of bi-coloured pixels for H&E staining. The authors thus propose to extract new axes in this plane, as detailed in the next section. Li *et al*.^11^ propose to use the NMF approach with a specific initialisation for H&E staining to avoid bad local minima. Without detailing the initialisation step Xu *et al*.^16^ recently introduced sparse non-negative matrix factorization (SNMF) for H&E and IHC staining, compared it to PCA, ICA and NMF and conclude that SNMF is better.

Table 1 summarises three image normalisation methods based on blind colour deconvolution to which we provided some adaptations as detailed later to finally obtain 5 different normalisation methods to test for IHC images. Table 1 indicates that the methods use optical density (OD) transformation of the RGB images before colour deconvolution. OD is defined by means of equation (1).


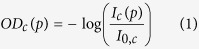


where *I*_*c*_(*p*) is the image intensity of pixel *p* in linear RGB channel *c* and *I*_0,*c*_ is the background intensity in the same channel. This transformation enables a first normalisation step with respect to background intensity usually labelled *I*_0_ which is supposed to correspond to the glass slide in the digital pathology context. However, the *I*_0_ computation methods which are based on maximum intensity, as mentioned in Table 1, do not ensure a correct extraction of the glass side intensity and can thus be improved (cf. next section). The use of an OD model for DAB staining is debatable because of its known scattering behaviour. However, Matkowskyj *et al*.^4^ show that a relative log-linearity exists between the DAB signal obtained in standard IHC and the antigen amount. Finally, OD-like DAB measurements have shown interesting properties for colour deconvolution and the quantification of the DAB signal^5^,^16^,^17^. It is these properties that we use in the present study.

In a preliminary study^18^ we adapted image normalisation methods from refs 8 and 11 to IHC staining. In the present study we include the SNMF-based method proposed in ref. 16 and notice the strong impact of initialisation on the algorithm performances in the IHC context. While some image normalisation methods^11^,^14^,^16^ stop after colour matching, others^8^,^15^ have an additional step of distribution fitting of deconvoluted values. This step aims to offset additional (counter)stain intensity variations from one staining batch to another. However, the actual contribution to normalisation of this step was not evaluated. In the present study we quantitatively assess the actual need of this additional step in the case of IHC staining. Finally, while some studies^15^,^16^ provide quantitative validation for H&E image normalisation, at least for those mentioned in this section, none provide actual quantitative evaluation for IHC image normalisation. In the present study, we propose an experimental framework based on TMA materials, image processing and statistical analyses to objectively evaluate the need for image normalisation and to identify the best way to perform it. This methodology should be viewed as a preprocessing step to include in the workflow before the use of any image analysis software. Indeed, after the identification of the best normalisation method to apply, new, normalised, images can be produced in an appropriate format (e.g., NDPI Hamamatsu proprietary format or any other, such as BMP) to be then submitted to a quantification software (commercially available, open source or developed using Matlab, Python, …).

## Methods

### Methodology overview

TMA is a widely used histological technology where small samples, a.k.a. cores, are extracted from different paraffin-embedded tissue blocks using a needle and inserted into a new paraffin block. A TMA slice thus enables the IHC analysis of numerous tissue samples at once. Subsequent slice imaging permits the extraction of data distributions characterising the IHC experiment or batch. In our methodology, a slice from a reference TMA is included in each IHC batch in order to provide comparable staining references (see Supplementary Fig. S1a–d). After digitisation at 20x, the TMA images are used to provide different data sets:

- To establish blind colour decomposition for each IHC batch;
- To statistically characterise the possible inter-batch variations and thus evaluate the need for image normalisation;
- To choose the best way to normalise IHC images, if required;
- To establish the transformations required to normalise the complete series of IHC images.

**Figure 1:**
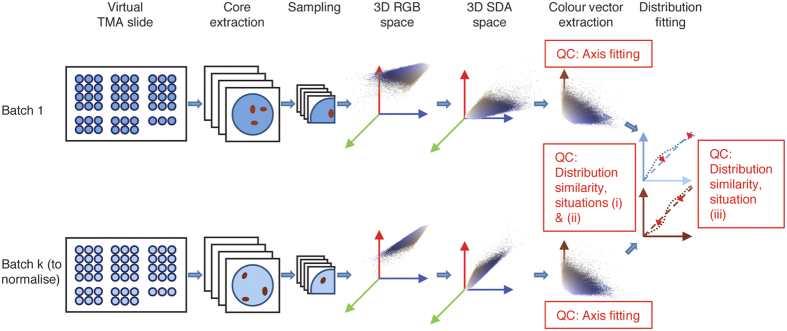
Overview of our methodology: it is based on the processing of virtual TMA slides to assess the need and the efficiency of IHC image normalisation methods (see main text for details). SDA: staining darkness (cf. section “Colour vector extraction and matching”). QC: quality control step (cf. section “Need and efficiency of the image normalisation steps”).

Figure 1 schematically presents the different steps of our approach which are detailed in the following subsections. Details on the software packages and the hardware used are provided as supplementary information.

### Reference image sampling

We use a method that we previously developed for processing whole TMA slide images in order to correctly identify the numerous circular tissue samples (600 μm-diameter cores) present on the slide^19^. To ease and to speed up the setup of the normalisation process we create a tiled image from each TMA image characterising an IHC batch, as illustrated in Supplementary Fig. S1e. This is made on each full resolution TMA slide image (0.452 μm/pixel) by cropping at a specific location of each core a square area of around 250 μm large (i.e. 500 × 500 pixel area). Since our TMA slide images count about 100 cores, each batch-reference image includes about 25 million pixels. To test the method robustness we generate different tiled reference images per IHC batch for each marker analysed, by systematically cropping different core locations. We subsequently investigate the possibility to reduce the number of TMA cores.

### Colour vector extraction and matching

This step aims to extract the colour vectors representative of the stains routinely used in IHC, i.e. brown for DAB-stained tissue evidencing protein expression and blue for HEM tissue counterstaining. In the normalisation methods summarised in Table 1, colour vector extraction is based on deconvolution after that each (RGB) channel intensity is transformed into OD (see equation (1)). As mentioned in the state of the art, an OD-like transformation of staining intensity has various advantages even if the Beer-Lambert law does not apply. To avoid misinterpretation we introduced the terminology of “staining darkness” (*SDA*) for this quantity.

#### Staining darkness transform

We compute *SDA* using equation (2).


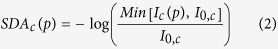


where *I*_*c*_(*p*) is the image intensity of pixel *p* in linear RGB channel *c* and *I*_0,*c*_ is the background intensity in the same channel. In the present study we improve the way to compute *I*_0_ by explicitly quantifying the intensity of the glass slide background. To this aim, we transform the tiled image into a greyscale one by selecting for each pixel the minimum *I*_*c*_(*p*) value among the RGB channels. We apply Otsu’s thresholding on this greyscale image to detect the tissue and a morphological dilation is applied to form a tissue mask. To avoid outliers, which could be due to glass artefacts, we set *I*_0,*c*_ at the mode of the intensity distribution in each channel outside this mask. We introduce the *Min* function in equation (2) to avoid any negative value (due to very light pixels for which *I*_*c*_(*p*) > *I*_0,*c*_). We apply this *SDA* transform before the extraction of colour vectors regardless of the used method, contrary to what we did in our preliminary study^18^, where the different OD transforms mentioned in Table 1 for refs 8 and 11 were used and exhibited some flaws.

#### Blind colour deconvolution

We test five different methods to extract the colour vectors in the 3-dimensional (3D) *SDA* space. All of them are based on the factorization of an *SDA* data matrix, **X** (3 × *n*), as in equation (3). However, they essentially differ by their choice of the objective function and/or constraints, as below:


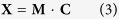


where **M** (3 × 3) is the colour deconvolution matrix and **C** (3 × *n*) the deconvoluted coefficient matrix.

In the context of standard IHC images **M** is defined on the basis of colour vectors defining the blue (HEM) and brown (DAB) axes, as shown in equation (4).


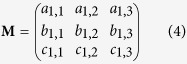


where **a** and **b** are normalised vectors defining the blue (HEM) and brown (DAB) axes, whereas **c** is their cross product. In fact, this 3^rd^ vector is not absolutely required but can be used as quality control (for which very small values are expected) for the HEM-DAB plane. In addition, visualisation of the pixel clouds in the 2D deconvoluted space easily reveals the defects of a deconvolution method, as illustrated in the results (see section “Colour vector extraction”).

**Figure 2:**
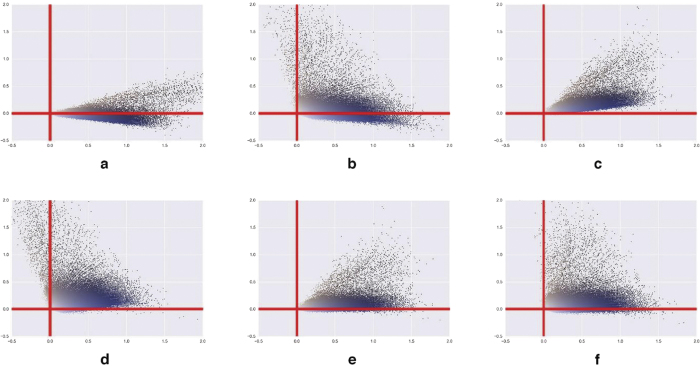
ERG experiment: pixel clouds on HEM (X) - DAB (Y) plane determined by means of (**a**) PCA, (**b**) “Li-init”, (**c**) “Li-init-NMF”, (**d**) “Macenko”, (**e**) “SNMF” (with standard initialisation), (**f**) “Li-init-SNMF”. The scatterplots show only 2500 colored pixels regularly sampled from the reference images.

**Figure 3:**
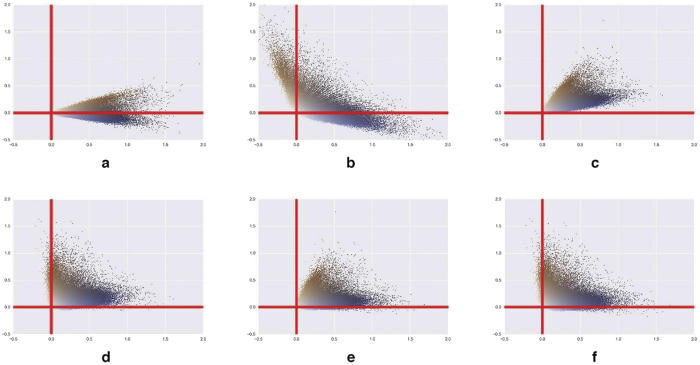
IGF2R experiment: pixel clouds on HEM (X) - DAB (Y) plane determined by means of (**a**) PCA, (**b**) “Li-init”, (**c**) “Li-init-NMF”, (**d**) “Macenko”, (**e**) “SNMF” (with standard initialisation), (**f**) “Li-init-SNMF”. The scatterplots show only 2500 pixels.

**Method 1**, **labelled** “**Macenko**”, was adapted from ref. 8. This method is based on the fact that the IHC images are two-toned and the pixel colour distribution in the SDA space should be mainly located in a plane, which can be identified by PCA. This hypothesis can be easily verified by computing the percentage of explained variance, which nears 100% in all our experiments. However, the orthogonal constraint makes principal components (PCs) inappropriate for the description of pixel colours, as illustrated in Figs 2a and 3a, where the two PCs were translated to the origin to preserve the SDA system origin. New axes have thus to be determined on the principal plane to better fit the colour cone. As detailed in the supplementary information, the new blue and brown axes are defined on the basis of the angle between each pixel and the 1st principal component, the x-axis in Figs 2a and 3a. To compute the deconvolution matrix (**M** in equation (3)), these two colour vectors are normalised, with the 3^rd^ vector as their cross-product.

**Methods 2 and 3**, **respectively labelled** “**Li**-**init**” **and** “**Li**-**init**-**NMF**”, are adapted from ref. 11. We separately consider the two steps of the deconvolution method proposed in ref. 11 for an extension described in Method 5. The NMF approach aims to avoid negative values in the final deconvoluted plane, as illustrated in Figs 2b,c and 3b,c. However, NMF is subject to local minimisation that is initialisation-dependent and thus requires a good approximation of the colour vectors to initialise the factorization process. The initialisation step (labelled “Li-init”) involves a clustering step into the HSV colour space to extract the two initial colour vectors, which are converted back to RGB and then to SDA in the IHC context. To extract the final vectors, an NMF algorithm is then used. We call this complete process “Li-init-NMF” (see details in the supplementary information).

**Methods 4 and 5**, **respectively labelled** “**SNMF**” **and** “**Li**-**init**-**SNMF**”. Xu *et al*.^16^ propose to use SNMF in place of NMF to extract the colour vectors. SNMF differs from NMF by its objective function in which regularisation and sparsity terms are introduced (see supplementary information) to help circumvent problems that can be found with NMF. However, the authors give no information about the initialisation they use for the SNMF algorithm. In our experiment, we test this method with a standard initialisation described in ref. 20 or proposed in ref. 11. These two variants are respectively labelled “SNMF” and “Li-init-SNMF”.

Because of the time needed for processing the factorization methods based on NMF and SNMF, it should be noted that we only use 0.1% of the pixels regularly sampled from our reference image, i.e. around 25,000 pixels per TMA, to extract the colour vectors representative of an IHC batch. With regard to this sampling, the robustness of the colour vectors so extracted was also analysed (see section “Colour vector extraction”).

#### Inter-batch colour matching

After obtaining deconvolution matrix **M** of equation (3) by one of the above methods, we can decompose an SDA image into their stain components (DAB and HEM in our IHC context). In our methodology the **M** matrix is independently computed on each SDA-transformed tiled image that we use as IHC batch reference. Thus, for batch *k*, the SDA-transformed tiled image can be deconvoluted by applying equation (5) with its own colour deconvolution matrix (**M**_*k*_).


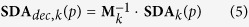


After applying deconvolution on each tiled image, the extracted colour vectors define the (HEM-DAB) colour space specific to each IHC batch. If the deconvolution is appropriate and efficient, the pixel vectors, **SDA**_*dec,k*_, should be in similarly aligned colour spaces and thus provide comparable value distributions for subsequent image processing.

### SDA distribution fitting

This step takes place per deconvoluted channel (HEM or DAB) and aims to accurately match the **SDA**_*dec*_ value distributions extracted from the tiled images representing the different IHC batches. A batch, labelled *t*, is chosen to be the target to which each other batch, labelled *k*, will be fitted. This additional step is not always considered in the different methods encountered in the literature. We test four fitting methods, including two techniques proposed in the literature^8^,^15^ and two generalisations that we introduce, i.e. linear and B-spline regression methods (see details in supplementary information).

Each fitting method defines a transform to match the *SDA*_*dec,k,c*_ distribution with the target (*SDA*_*dec,t,c*_) one. The result is labelled Fit_*t*_(*SDA*_*dec,k,c*_) and the corresponding vector, **Fit**_*t*_(**SDA**_*dec,k*_), expresses the normalised SDA values of a pixel in the deconvoluted space.

### Need and efficiency of the image normalisation steps

We remind that in the proposed methodology a slice from the same TMA block is added to each IHC batch. In the context of our controlled platform (see section “Experimental design for quantitative evaluation”), we can assume that for these slices the distributions of the *SDA*_*dec*_ values should be similar in the absence of inter-batch variations. Accordingly, to evaluate the quality and the actual contribution of the different normalisation steps, we evaluate the similarity between the *SDA*_*dec,t,c*_ and the *SDA*_*dec,k,c*_ value distributions in the three following situations: (i) before colour matching, i.e. by imposing the colour vectors extracted from batch *t* to each batch *k*, (ii) after colour matching between the batches and (iii) after an additional SDA distribution fitting step (see QC steps in Fig. 1). In each situation the distributions we compare are established from pixel samples different from the tiled images which are used both for colour vector extraction and to establish an additional SDA transform for improving distribution fitting. Distribution similarity is then checked by means of Q–Q plots established on 1000 quantiles of each distribution. These plots should fit the 45-degree reference line (*y* = *x*) in the case of distribution equality. We evaluate this fitting property in terms of the root-mean-square error (RMSE). We also use the 2-sample Kolmogorov-Smirnov (KS) statistic which computes the maximum difference between the empirical cumulative distribution functions of the two samples. In practice, only the step(s) actually contributing to batch distribution similarity should be applied.

### Image normalisation

Before staining quantification (see section “Experimental design for quantitative evaluation”) we apply the inter-batch normalisation process on the complete TMA images as follow:

1. Converting each image to stain darkness using equation (2).
2. Applying the deconvolution using the vectors extracted from the tiled reference image using equation (5).
3. Optional: applying the SDA fitting step according to the target to each pixel of the image.

While the first two steps constitute an intra-batch process generating a normalised (and thus comparable) colour space, the third step operates inter-batch to provide single-channel value distribution mapping. This third step requires choosing one batch (e.g. the first one) as the target to which all the others should be adjusted. Once this is done, the staining quantification can be carried out in the deconvoluted space. For visualisation purposes, enabling among other qualitative evaluation and/or biomarker scoring by pathologists, it is possible to go back to a normalised RGB image using equations (6) and (7), which respectively compute the normalised (*N*) SDA vector and the normalised intensity values in the RGB space, using equation (2), for images of batch *k* taking batch *t* as target.


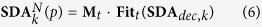



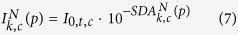


## Experimental design for quantitative evaluation

We carried out different IHC staining experiments to provide images for quantitative analyses. These experiments targeted different proteins which show different subcellular localisations and were evidenced in different tissue types, as detailed in Table 2.

To evaluate the normalisation methods each IHC experiment was done in different staining batches in which we included consecutive slides (one per batch) from the same TMA in order to screen the targeted protein expression (see Table 2). All tissue samples analysed in this study came from the archives of the Department of Pathology of the Erasme University Hospital. All patients gave informed consent and the study was approved by the local ethics board. For each marker the same IHC protocol (with the same primary antibody at the same dilution detailed in the supplementary information) was used, which is a minimum commonly admitted condition for standardising IHC. It should be noted that contrary to the other makers, the two CD3 batches were carried out with two different lots for the antibody as well as for the detection kit. This experiment enabled us to test the robustness of our approach in a more realistic context.

The whole TMA slides were then digitalized at 20x just after IHC staining using a calibrated whole slide scanner (Nanozoomer, Hamamatsu, Hamamatsu, Japan). The calibration concerns light intensity, white balance and shading and is done every day automatically, using a specific slide provided by the manufacturer. A second scanning was also performed later for some slides, as detailed in the result section. After scanning, one TMA image, e.g. from the first batch, was chosen as the target image and the other TMA images from the other batches were submitted to the different steps of the normalisation and quality control methods to fit the colour and SDA distribution characteristics of the target, according to Fig. 1. This process enabled us to select normalisation methods providing satisfactory results at SDA distribution levels.

Finally, we analysed the impact of the selected normalisation methods on two features commonly used by pathologists to characterise IHC staining, i.e. the Labelling Index (LI), which is the percentage of positive (DAB-stained) tissue area or nucleus area (for nuclear markers), and the Quick Score (QS), which is the product of the LI by the mean SDA computed on the positive (DAB-stained) tissue/nucleus pixels only, i.e. this is the mean SDA where the negative tissue/nucleus pixels have zero-setting values^21^. To compute these quantitative features, three or, in the case of nuclear markers, four segmentation parameters were manually set in the deconvoluted (HEM-DAB) plane obtained for the target batch only. We noticed that in this plane the tissue pixels have a distance to the origin larger than a given threshold, i.e. located outside of a disk centred at the origin, and positive protein staining consists of pixels for which their brown value exceeds both a given threshold and their blue value by a given factor. In the cases of nuclear markers a fourth parameter consists of an additional threshold on the blue values to identify negative nucleus area^21^. As our image acquisition process is standardised, the value of the tissue threshold can be set at a small value arbitrarily chosen at 0.015. The remaining parameters determining positive DAB staining (and negative nuclei if required) were set by a pathologist. An efficient image normalisation should avoid these parameters to be adjusted between the different batches. The segmentation parameters established for the target batch were thus used for all the batches, to extract the LI and QS values from each tissue core found in each TMA slide. We thus obtained LI and QS distributions extracted per protein and per batch (on about a hundred of cores per TMA slide). As we used consecutive slides from the same TMA, after image normalisation the LI and QS distributions characterising a given protein should be similar from a batch to another. This property at distribution level does not assume concordance between the pairs of values measured on two slices of the same TMA core. To evaluate distribution fitting we used the 2-sample KS test, which uses the KS statistic described in section “Need and efficiency of the image normalisation steps” to evaluate the null hypothesis stipulating the equality of probability distributions.

## Results

We first present the results which concern the first 4 markers of Table 2 because they were revealed in similar conditions, i.e. the different IHC batches were carried out with the same lot of antibodies and reagents. Finally, we describe the results obtained for the CD3 experiment to evaluate the robustness of our approach when different lots of antibody and reagents are used. In this latter section, we also report data concerning robustness with regard to the number of TMA cores required to extract an efficient normalisation transform.

### Evaluation of colour deconvolution methods

#### Colour vector extraction

We assessed the ability of each method to extract relevant colour axes from a reference image. Figures 2 and 3 compare the results of the different deconvolution methods under study applied on an ERG and an IGF2R staining batch, respectively. It should be noted that in comparison to IGF2R, the ERG experiment exhibited strongly unbalanced proportions of brown and blue pixels. This is due to the fact that the nuclear ERG staining is less present than the cytoplasmic/membranous IGF2R one, as illustrated in Fig. S1a,b. Figures 2 and 3 also clearly show that two methods (“Macenko” and “Li-init-SNMF”, cf. frames d and f) provide interesting solutions in terms of colour vector extraction. It should be noted that “Macenko” has a controlled amount of negative values for the training data (1% of non-background pixels for each deconvoluted channel). These negative values can be considered as indicators of a negligible contribution to the concerned channel and can thus be clipped to 0. It is interesting to note that the SNMF-based methods have smaller numbers of negative values than the “Macenko” method for the HEM channel only, whereas larger amounts of negative values (i.e. about the double) are observed for the DAB channel, while clearly improving the results obtained after the “Li-init” initialisation step (in Figs 2 and 3).

To confirm all these observations, we carried out different measurements to characterise the DAB and HEM axis fitting to the pixel clouds and the axis robustness to the change of the tiled reference images used for vector extraction. These analyses were made on 3 staining batches of the ERG and IGF2R experiments, with 3 different tiled reference images per batch. We assessed the quality of the plane determined by the two colour axes by analysing the values obtained on the 3rd orthogonal axis and obtained near identical results for all methods, except “Li-init”. These similar results show very low contributions to the 3rd dimension and no noticeable variation induced by the tiled reference image. As expected, the planes extracted by “Li-init” appeared a little less suitable and a little less robust. However, this method provided good initial planes for further optimisation. These analyses also confirmed that the HEM axis extracted by the “Li-init-NMF” method and the DAB axis extracted by both “Li-init-NMF” and “SNMF” methods did not sufficiently fit the pixel clouds, as observed in Figs 2 and 3 (frames c and e). We thus confirm the selection of the “Macenko” and “Li-init-SNMF” methods, together with “Li-init” as an interesting initial step, and discard the other methods from the subsequent analyses because of their obvious defects.

**Figure 4:**
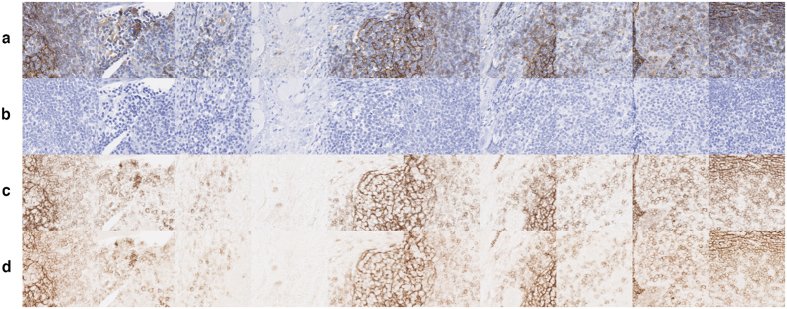
Fields from a tonsil tissue slide where CD21 expression was revealed by means of IHC. (**a**) DAB staining with HEM counterstaining. (**b**,**c**) HEM and DAB channels extracted from (**a**) using “Macenko”. (**d**) DAB staining of (**a**) scanned before counterstaining (i.e. DAB ground truth).

To illustrate the relevance of the extracted colour vectors, we carried out an experiment using a tonsil tissue slide submitted to IHC targeting CD21 (a transmembrane protein). The same tissue slice was scanned before and after HEM counterstaining resulting in two images: one with “pure” DAB staining and the other with both staining and counterstaining, respectively. Fifteen tiles (870 × 980 μm at 10x) were picked from this latter and used to constitute one tiled image from which colour vectors were extracted using the “Macenko” deconvolution method. Figure 4 illustrates the deconvolution results obtained after going back to RGB images using equations (6) and (7).

#### Inter-batch colour matching

**Figure 5:**
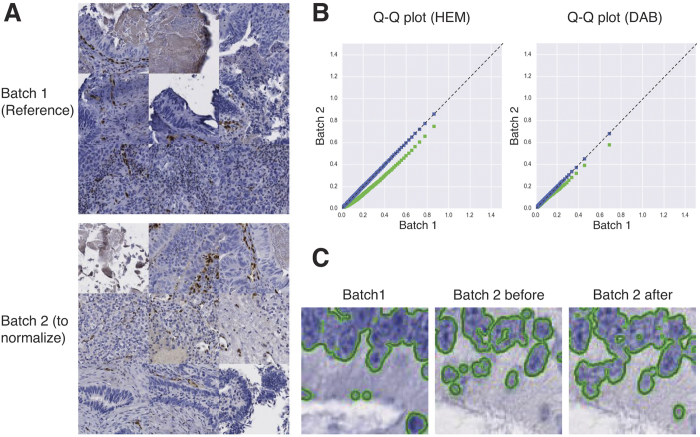
Effects of colour matching on HEM and DAB SDA distributions and on nucleus area segmentation for the ERG experiment. (**A**) Several tiles extracted from the TMA cores characterising two IHC batches targeting the ERG protein and showing lighter staining in the second batch, in particular for HEM. (**B**) Q-Q plots (X: reference batch, Y: batch to normalise) comparing the SDA distributions measured in the HEM and DAB channels before (i.e. with the colour axes computed on batch 1, in green) and after colour matching (i.e. after matching the colour axes computed on each batch, in blue). Lighter colours in batch 2 make the Q-Q plots under the diagonals before matching. (**C**) Zoomed images from the two batches with the segmentation of the negative nucleus areas (HEM staining), using the parameters set for batch 1, before and after colour matching. Because of the lighter blue staining in batch 2, some nucleus parts are lost for this batch before colour matching whereas they are recovered after colour matching.

We then analysed the efficiency of each of the three methods selected above when used for inter-batch colour matching. For this purpose, we characterised for each marker and each deconvoluted channel (HEM and DAB) the similarity of the two SDA distributions (from two batches) obtained before and after colour matching. In the first case, the colour vectors were extracted from the reference batch only and imposed to the second batch. In the second case, the colour vectors were extracted from each batch and matched. For robustness purposes we made our analyses on pixel samples different from the reference images used for vector extraction. Figure 5 illustrates the Q–Q plots of the SDA distributions obtained for ERG before and after colour matching based on the “Macenko” deconvolution method. This figure shows the improvement in fitting the 45-degree reference line after colour matching, especially for the HEM channel. Table 3 provides the quantitative results obtained for ERG and IGF2R, where the distribution similarities are evaluated by two criteria. The first one (RMSE) evaluates the fitting of the Q–Q plot with the 45-degree reference line (*y* = *x*), whereas the second is the two-sample KS statistic. For the HEM channel the results show a strong improvement of the distribution similarities after colour matching that is evidenced by a decrease near 0 of each criterion and for each method. In particular, the KS statistic, which shows a maximum difference of about 20% between the two cumulative distribution functions before matching, decreases until 2–3% after matching. In contrast, the DAB channel shows a strong stability between the two batches, as revealed by the very low criterion values already obtained before colour matching. This means that the DAB vector extracted from the 1^st^ batch is also adapted for the second one. However, few improvements can be observed in terms of the KS statistic for ERG, especially for “Li-init” which was not as good as the others before matching (also observed in the case of IGF2R). The results for PDFRa and P53 exhibit different behaviours, as detailed in Table 4. Indeed, PDGFRa shows improvements for both the HEM and the DAB channels. Concerning DAB, the KS statistic for “Macenko” and “Li-init + SNMF” decreases from 10% to about 2.5%, whereas for HEM, this statistic decreases from 13% to about 5% for these two methods. Finally, the results obtained for P53 show opposite data as compared to ERG. Indeed, this time the HEM channel shows inter-batch stability (KS statistic around 4% before colour matching), whereas the DAB channel shows improved distribution similarities after colour matching, with a decrease which amounts to between 14 and 20% in terms of the KS statistic, paralleled by a decrease in terms of RMSE.

### Contribution of an additional SDA distribution fitting step

In view of the very good results already obtained after colour matching, we observed no additional improvement after SDA distribution fitting whatever the method used. It should be noted that the IHC experiments were carried out with the same antibody and reagent lots and in a standardised environment, consisting of automated slicing and IHC process, image acquisition using a calibrated whole slice scanner. Our results thus show that in these conditions the inter-batch variations essentially affect the colours without additional effect on the staining intensity. It should even be noted that the P99-based rescaling proposed in ref. 8 for H&E staining causes a systematic degradation of the fitting obtained after colour matching (for the 4 markers) and should thus be absolutely avoided in the case of IHC staining.

**Figure 6:**
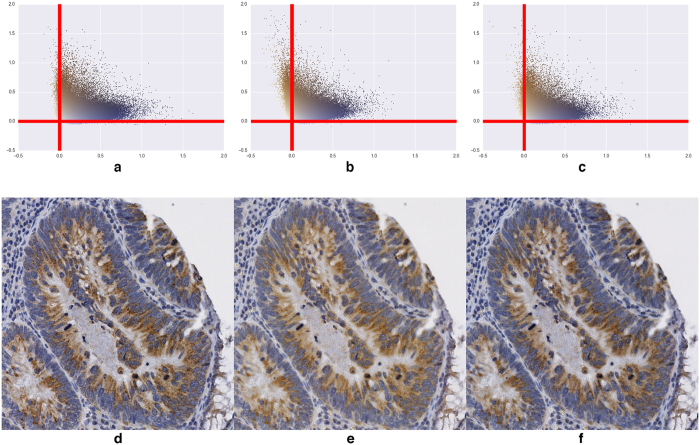
Application of colour matching (with “Macenko”) to reduce the effects of colour fading. (**a**) Pixel cloud from an IGFR2R slide using the colour vectors extracted from its reference image. (**b**) Pixel cloud of the same slide scanned 7 months later using the colour vectors of (**a**). (**c**) Pixel cloud of (**b**) with the new colour vectors extracted with “Macenko”. (**d**–**f**) Part of a core from the TMA image corresponding to the pixel cloud in (**a**–**c**) respectively.

We carried out an additional experiment to investigate whether the fading of IHC staining on tissue slides (in the course of time) has no additional effect on intensity. To this aim, we scanned a second time, 7 months later, the TMA slides of the IGF2R and ERG experiments. Again, the essential effect was observed at the colour level, as illustrated in Fig. 6 for IGF2R. Indeed, we observed a yellowing of the stains 7 months later, resulting in frame b (before colour matching) in a misalignment of the pixel cloud to the axes extracted in frame a. After colour matching with the new axes extracted from the image obtained 7 months later (using “Macenko”, see frame c), the KS statistic between the SDA distributions for IGF2R (comparing frames a and c of Fig. 6) was 1% for HEM and 2% for DAB (against 12% and 6%, respectively, before colour matching when comparing frames a and b). For ERG it was 4% for HEM and 5% for DAB (against 24% and 10% before colour matching, respectively). Again, the very good results obtained after colour matching made unnecessary an additional distribution fitting step.

### Application to IHC biomarker quantification

**Figure 7:**
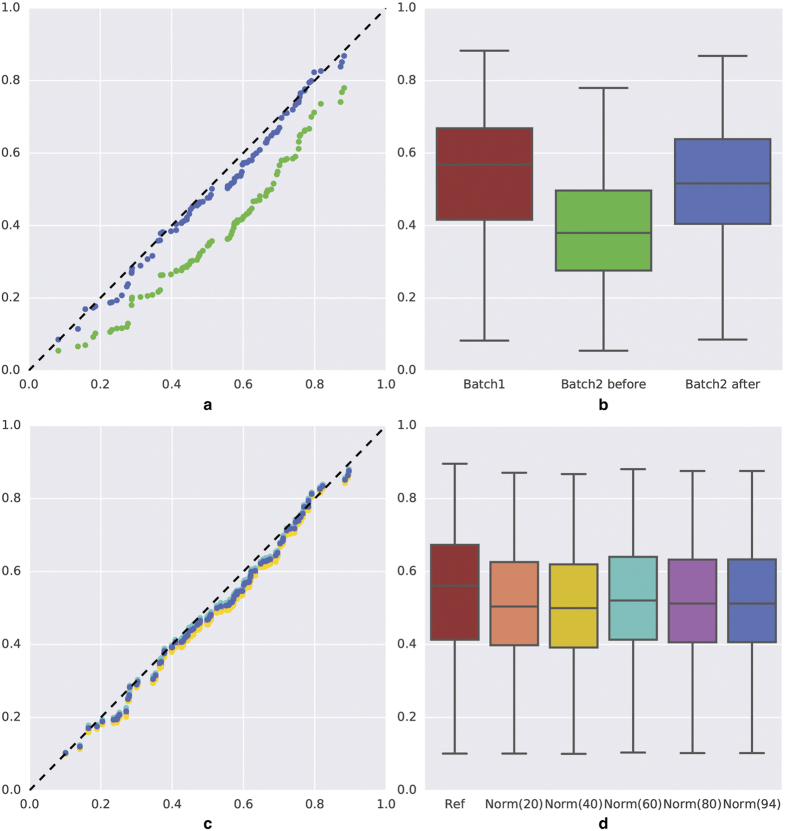
Effects of image normalisation on the quantification of the PDGFRa labelling index (LI). (**a**) Q-Q plots (X: reference batch, Y: batch to normalise) comparing the LI distributions before (in green) and after (in blue) colour matching between two IHC batches targeting PDGFRa. (**b**) Box plots of the PDGFRa LI computed in the different situations indicated on the X-axis. Box boundaries = 1st and 3rd quartiles, inner horizontal line = median, whiskers = minimum and maximum values. (**c**,**d**) Similarly, illustration of the impact of the number of cores (mentioned on the X-axis of frame (**d**)) selected in the TMAs to normalise the second PDGFRa batch with regard to the first one (ref). The dot colours in (**c**) correspond to those of boxes in (**d**).

To evaluate image normalisation usefulness for quantitative IHC staining characterisation, we extracted two features commonly used by pathologists, i.e. the Labelling Index (LI) and the Quick Score (QS). We analysed their distributions extracted from the TMA slides as detailed in section “Experimental design for quantitative evaluation”. Figure 5 illustrates the problem observed for nucleus area segmentation before colour matching and the improvement obtained after this step. Tables 5 and 6 summarizes the quantitative results obtained (for colour matching without additional SDA distribution fitting) for “Macenko” and “Li-init-SMNF”, i.e. the two methods showing the best performances and robustness in the previous sections. These two methods provided very similar results, in agreement with the results obtained in terms of SDA distributions in section “Inter-batch colour matching”. Before colour matching the KS test showed significant variations between the two LI distributions for each marker, except IGF2R. For the QS feature a substantial gain in terms of the KS statistic was nevertheless obtained for this marker also (Table 6). After colour matching, all the variations were reduced to non-significant values. These KS values were larger than those obtained for the SDA distributions in section “Inter-batch colour matching” because the present data were computed on samples 10 times smaller than in Tables 3 and 4. Figure 7a,b illustrates the inter-batch variations observed on the LI distributions for PDGFRa before image normalisation and the distribution similarity obtained after colour matching. Confirming our results obtained at the SDA distribution levels (see section “Contribution of an additional SDA distribution fitting step”), adding an SDA distribution fitting step did not improve the results and even deteriorated them often, in particular with the P99-based rescaling proposed in ref. 8.

### Robustness regarding the changes of antibody and reagent lots and the number of TMA cores

#### Changes of antibody and reagent lots

**Figure 8:**
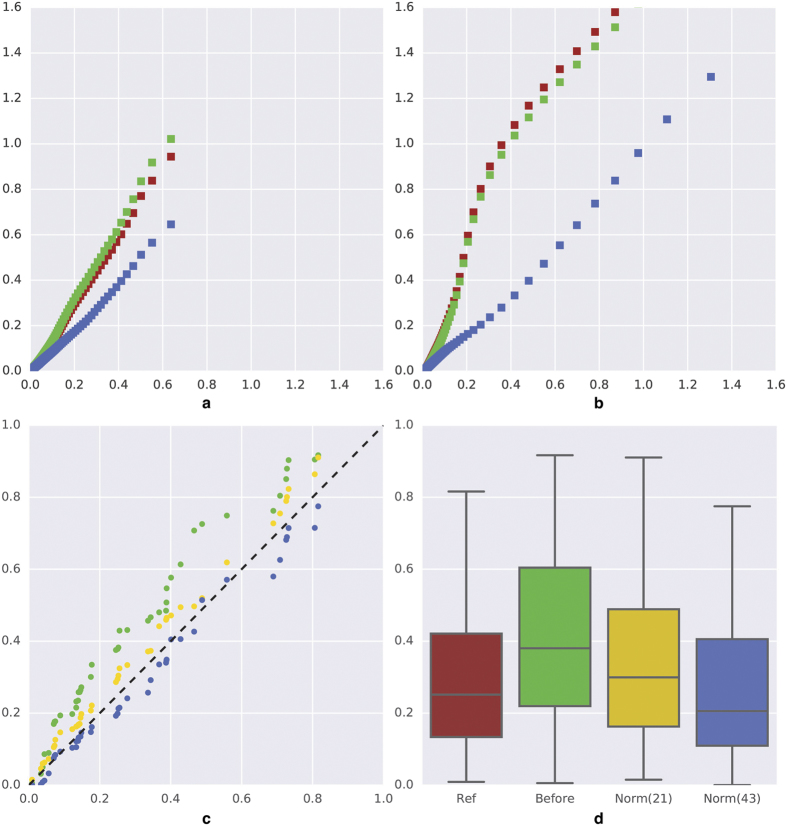
Effects of image normalisation for the CD3 experiment. (**a**,**b**) Q-Q plots (X: reference batch, Y: batch to normalise) comparing the SDA distributions measured in the HEM and DAB channels, respectively, before colour matching (in green), after colour matching (in red) and after additional distribution fitting (in blue) using 100 B-splines (cf. supplementary information). Darker colours in batch 2 make the Q-Q plots above the diagonals before normalisation. (**c**) Q-Q plots (X: reference batch, Y: batch to normalise) comparing the LI distributions after batch 2 normalisation using 21 (in yellow) or 43 TMA cores (in blue). (**d**) Box plots of the CD3 LI computed for batch 1 (ref) and batch 2 before and after normalisation based on the use of either 21 or 43 TMA cores. The associated KS-statistics (p-values) are 0.19 (0.39) and 0.12 (0.91), respectively. Box boundaries = 1st and 3rd quartiles, inner horizontal line = median, whiskers = minimum and maximum values.

We applied exactly the same methodology for the CD3 experiment than for the other markers. It should be noted that for CD3 the TMA only counted 43 cores (i.e. less than 50% compared to the other TMAs). As illustrated in Supplementary Fig. S2, our methodology evidenced that the changes of lots between the two CD3 batches strongly impacted the SDA values distributions of the second IHC batch (in particular the DAB one, with an RMSE of 0.35 before normalisation). This impact was not corrected by means of colour matching and thus required additional SDA distribution fitting. The best result was obtained by using B-spline regression (with 100 splines). This method was particularly efficient in correcting the non-linear deformation of the Q-Q plots shown in Fig. 8b (RMSE of 0.04 after normalisation). Supplementary Table S2 confirmed the positive impact of this normalisation on the quantitative LI and QS features characterising CD3 expression. These data show a decrease of the KS statistics from around 50% to reach clearly non-significant values.

#### Number of TMA cores

Finally, we tested how our method was affected by the number of TMA cores used to extract the normalisation transform. Figure 7c,d shows the variations observed on the PDGFRa LI distribution, with the largest inter-batch variations in Table 5, when the number of cores progressively increase from 20 (regularly selected in the TMA grid) to the maximum, 94. It should be noted that the TMA samples were from a single tissue type (glioblastoma, see Table 2). The results show that 20 cores were enough to identify an efficient transform and no improvement was observed from 60 cores. We replicated the same test on the ERG experiment for which the TMA was constituted of nine different tissue types. To follow the tissue origin distribution we first selected a set of 3 cores per tissue type that we progressively completed. As detailed in Supplementary Fig. S3 the results show that the first selection of 27 cores is enough to extract an efficient normalisation transform. In the case of CD3 (see Fig. 8c,d and Supplementary Fig. S2) a selection of 21 cores, which follow the tissue origin distribution, improves the KS statistic from 29% (before normalisation, see LI feature in Table S2) to 19% (p = 0.39). The use of 43 cores improves it up to 12% (see Table S2). Another core selection, which did not take into account tissue origins, provided unsatisfactory results (KS statistic of 26%).

## Discussion

In their review Onder *et al*. highlight the need for comparative analyses between image normalisation methods in histopathology^22^. They stress the facts that just showing visual improvements is not sufficient and that quantitative evaluation and comparison are required. The present study proposes an experimental framework to perform this comparison in the context of IHC applied to various markers and tissue types. Regarding SDA and biomarker feature values, the use of TMA slides sliced from the same TMA block provides reference distributions which should fit after efficient image normalisation. We are thus left with measuring distribution fitting before and after the different steps of image normalisation to evaluate their actual impact and necessity.

We apply our experimental framework in the IHC context to evaluate different image normalisation methods based on blind colour deconvolution. Our experimental analysis reveals that efficient colour deconvolution is an essential step which has to be applied on each staining batch. This means that colour vectors extracted from an IHC slide series cannot be applied as is to another series, even between successive IHC batches made in the same and well-controlled environment (such as illustrated by our results labelled “before”). Consequently, any IHC quantification tool based on colour deconvolution, provided in commercial or open-source software packages, must include such an adaptive step. This also means that the widespread use of the so-called “Ruifrok” method (i.e. the use of a single set of values, such as those given in ref. 17) cannot be satisfactory without adaptation to each slide series left to analyse.

Our analyses confirm results from a recent study showing that PCA and NMF are unsuitable for colour vector extraction^16^. However, we notice that the proposed SNMF alternative requires a good initialisation to be efficient. We adapt to IHC images the initialisation proposed by Li *et al*. (for the NMF method applied to H&E images^11^) and show that it is very useful to make an SNMF-based approach effective. This initialisation requires to transform the RGB colour space into the HSV one and then to come back to RGB before SDA transform. In our quantitative analysis the deconvolution method proposed by Macenko *et al*.^8^ provides results comparable to the “Li-init-SNMF” ones in terms of colour matching but with some practical advantages. Indeed, it is simpler to implement, does not require initialisation and provides control over the number of negative values. In contrast, the constraint balancing required by SNMF must be experimentally tuned, which is not always an easy task. However, the “Macenko” approach is designed for two stains only, without obvious generalisation for more stains. The SNMF method does not have this limitation as long as an adequate unsupervised initialisation can be provided.

In the literature the “Macenko” method is often denigrated as being less performing, at least with H&E staining, if one of the stains is less present (e.g., see ref. 12). Our IHC experiments included a nuclear marker (ERG) with relatively rare expression. In average, it represents about 10% of the total nuclear area, i.e. only about 3.5% of all the (counter)stained tissue area/pixels. Even if the DAB axis seems less well-aligned than in the case of a more abundant staining (see Figs 2 and 3), all the quantitative results regarding ERG are very satisfactory (cf. Tables 5 and 6). However, in the case of rare staining the DAB axis extraction can be improved by simply selecting a smaller quantile in the angle distribution (e.g. quantile 0.002 in place of 0.01).

In the well-controlled context of our laboratory, our data also show that an additional step of SDA distribution fitting may be unnecessary, even after colour fading in the course of time, when the same lots of reagents (antibodies and detection kits) are used across the IHC batches. In this case normalisation can be made from representative pixel colour samples, without the absolute need for including a slide from a common TMA in each series. For example, a tiled reference image can be directly sampled from the slide series in each batch. However, the property of SDA distribution stability after colour matching should be verified at least once in each context, in particular if the slides are processed in different laboratories with different operating procedures. As an illustration of a more general situation, the CD3 experiment was made with changes in the antibody and reagent lots between the batches and revealed the usefulness of an additional step of SDA distribution fitting. This kind of situation requires a tool to capture the SDA distributions in each batch. Slices from the same TMA block constitute a practical way for meeting this requirement with minimum additional cost (only one slide must be added per IHC batch). Biobanking currently eases tissue sample availability and tissue arrayer is an accessible technology and which we have had in our laboratory for about 10 years. In addition, TMAs, which cover a wide range of organs and disease states, are also commercially available.

The reported results show that our TMA-based methodology was able to correct linear and nonlinear (in the case of CD3) deformations of the (HEM or DAB) SDA value distributions. Our process successfully deals with deformations characterised by a KS statistic of 22% (HEM channel of ERG) or an RMSE of 0.35 (DAB channel of CD3). We strongly reduce the impact of the variability in slides from a single TMA block by working at distribution level and applying regression to a Q-Q plot, which does not require a pairwise matching of the cores between the TMA slides. The sole requirement is that the SDA value distributions can be extracted from the tiled images constructed from the reference TMA slides. This is the reason why a sufficient number of TMA cores are required. We notice that a TMA including around 20 cores is enough to normalise IHC images showing variations in terms of colour or SDA values. When tissue samples with different origins are investigated, we recommend to preserve this diversity in the TMA and, if need be, to increase the number of cores. If the targeted antigen expression is well known or documented, a selection of tissue samples in order to cover the staining intensity range from weak to strong is possible.

In our laboratory we currently apply the proposed normalisation process as a preprocessing step before performing quantitative analyses with a commercial program in the context of different research studies. So, the IGFR2R example shown in the paper is part of such study focusing on the role of this protein in tumour neovascularization and involving the quantitative evaluation of IGF2R expression in the vascular compartment of a large series of tumours (manuscript in preparation). In this evaluation we used the LI and QS features but the beneficial impact of image normalisation should also be observed for each staining feature which requires to detect positive and/or negative pixels, e.g. to segment the histological objects of interest. Such positive impact was already observed for object segmentation in the context of H&E staining^15^,^16^.

In conclusion, the present study enables us to provide guidelines for image normalisation applied to IHC staining when the expression pattern of a targeted protein should be analysed across a large slide series requiring multiple staining batches:

- When quantitative characterisation is concerned, a well-controlled platform for tissue processing, staining and image acquisition and standardised protocols are advised in order to reduce inter-batch variations.
- In the absence of prior information on inter-batch variations, it is advised to include a slice from a reference TMA in each staining batch in order to provide staining references in terms of colour and intensity, with a minimum of 20 cores with at least 3 cores per tissue origin.
- For two stains the colour matching step can be carried out using either the “Macenko” method or the “Li-init-SNMF” one. The use of the latter remains to be validated if more stains have to be discerned. Any other method proposed in the literature can be evaluated using the proposed methodology.
- The necessity of an additional SDA distribution fitting step and its type should be evaluated by looking at the SDA Q–Q plots obtained after colour matching and their deformation with regard to the 45-degree reference line. An RMSE larger than 0.05 requires evaluating the benefit of using this additional step. Non-linear deformation can be corrected by using B-spline regression.

## Additional Information

**How to cite this article**: Van Eycke, Y.-R. *et al*. Image processing in digital pathology: an opportunity to solve inter-batch variability of immunohistochemical staining. *Sci. Rep.* **7**, 42964; doi: 10.1038/srep42964 (2017).

**Publisher's note:** Springer Nature remains neutral with regard to jurisdictional claims in published maps and institutional affiliations.

## Supplementary Material

Supplementary Information

## Figures and Tables

**Table 1:** Summary of three image normalisation methods based on blind colour deconvolution.

Study	Staining	Preprocessing	Vector extraction for colour matching	Post-processing
Macenko *et al*.^8^	H&E	OD computing with *I*_0_ = *max(I*)	PCA & extraction of new axes	Rescaling OD distribution
Li *et al*.^11^	H&E	OD computing with *I*_0_ = *max(I*) after filtering	NMF with specific initialisation	None
Xu *et al*.^16^	H&E-IHC	OD computing (no detail on *I*_0_ computing)	SNMF	None

H&E: hematoxylin-eosin; IHC: immunohistochemistry; OD: optical density; *I/I*_0_: intensity/background intensity; PCA: principal component analysis; NMF: non-negative matrix factorization; SNMF: sparse NMF (for details see main text).

**Table 2:** IHC experiments (see details in supplementary information).

Marker	Antigen	Expression location	TMA composition
IGF2R (3 batches)	Insulin-like growth factor 2 receptor	Cell cytoplasm and/or membrane	Various healthy or pathologic tissue samples coming from different human organs
ERG (3 batches)	ETS-related gene	Cell nucleus	Various healthy or pathologic tissue samples coming from different human organs
PDGFRa (2 batches)	Platelet-derived growth factor receptor, alpha polypeptide	Cell cytoplasm and/or membrane	Various human glioblastoma samples
P53 (2 batches)	Tumour protein (or tumour suppressor) P53	Cell nucleus	Various human glioblastoma samples
CD3 (2 batches*)	Cluster of differentiation 3	Cell cytoplasm and/or membrane	Tonsil and colonic tissue samples

*Carried out with different antibody and reagent lots.

**Table 3:** Effects of colour matching on deconvoluted SDA value distributions for ERG and IGF2R (*n* = 1000).

Colour matching	ERG - RMSE		IGF2R - RMSE		ERG - KS		IGF2R - KS	
	Before	After	Before	After	Before	After	Before	After
HEM channel								
Macenko	0.084	0.013	0.061	0.013	0.218	0.022	0.197	0.031
Li-Init	0.106	0.036	0.087	0.016	0.220	0.020	0.197	0.021
Li-init-SNMF	0.096	0.015	0.066	0.015	0.219	0.023	0.197	0.032
DAB channel								
Macenko	0.030	0.025	0.009	0.011	0.057	0.032	0.046	0.049
Li-Init	0.021	0.024	0.023	0.016	0.103	0.026	0.105	0.059
Li-init-SNMF	0.019	0.018	0.007	0.010	0.033	0.032	0.022	0.057

Before: use of the deconvolution matrix extracted from batch 1.

**Table 4:** Effects of colour matching on deconvoluted SDA value distributions for P53 and PDGFRa (*n* = 1000).

Colour matching	P53 - RMSE		PDGFRa - RMSE		P53 - KS		PDGFRa - KS	
	Before	After	Before	After	Before	After	Before	After
HEM channel								
Macenko	0.028	0.022	0.044	0.028	0.042	0.076	0.132	0.047
Li-Init	0.036	0.017	0.067	0.062	0.045	0.076	0.093	0.059
Li-init-SNMF	0.034	0.015	0.046	0.023	0.048	0.077	0.135	0.051
DAB channel								
Macenko	0.039	0.008	0.059	0.015	0.167	0.028	0.107	0.025
Li-Init	0.037	0.020	0.073	0.034	0.220	0.029	0.055	0.056
Li-init-SNMF	0.034	0.010	0.057	0.016	0.192	0.015	0.103	0.027

Before: use of the deconvolution matrix extracted from batch 1.

**Table 5:** Comparison of the LI distributions before and after colour matching using “Macenko”/“Li-init-SNMF”.

LI	KS statistic		KS p-value		Mean - batch to normalise		Mean - target batch	SD - batch to normalise		SD - target batch
Marker	Before	After	Before	After	Before	After		Before	After	
IGF2R (*n* = 103)	0.09	0.06	0.81	0.99	0.17	0.18	0.18	0.15	0.15	0.15
ERG (*n* = 104)	**0**.**20**	0.1/0.12	**0**.**03**	0.70/0.47	0.14	0.12	0.10	0.11	0.09	0.07
PDGFRa (*n* = 94)	**0**.**37**	0.14	<**10**^−**3**^	0.31	0.39	0.51	0.53	0.18	0.19	0.19
P53 (*n* = 98)	**0**.**20**	0.17/0.14	**0**.**03**	0.09/0.25	0.22	0.20	0.21	0.22	0.25	0.26

The values obtained with the second method are indicated only when they are different from those obtained with the first method. SD: standard deviation.

**Table 6:** Comparison of the QS distributions before and after colour matching using “Macenko”/“Li-init-SNMF”.

QS	KS statistic		KS p-value		Mean - batch to normalise		Mean - target batch	SD - batch to normalise		SD - target batch
Marker	Before	After	Before	After	Before	After		Before	After	
IGF2R (*n* = 103)	0.14	0.06	0.28	0.99	0.06/0.05	0.06	0.06	0.06	0.07/0.06	0.07/0.06
ERG (*n* = 104)	**0**.**18**	0.12/0.13	**0**.**05**	0.47/0.37	0.11/0.08	0.10/0.07	0.08/0.06	0.09/0.07	0.08/0.06	0.07/0.05
PDGFRa (*n* = 94)	**0**.**30**	0.06	<**10**^−**3**^	0.99	0.18/0.17	0.25/0.24	0.26/0.25	0.10/0.09	0.12/0.11	0.12/0.12
P53 (*n* = 98)	**0**.**18**/0.16	0.16/0.14	**0**.**06**/0.13	0.13/0.25	0.16/0.13	0.16/0.13	0.17/0.14	0.23/0.20	0.25/0.21	0.25/0.22

The values obtained with the second method are indicated only when they are different from those obtained with the first method. SD: standard deviation.

## References

[b1] Moles Lopez X., Debeir O., Salmon I. & Decaestecker C. Whole slide imaging and analysis for biomarker evaluation in digital pathology. In Microsc. Adv. Sci. Res. Educ., 776–787 (Formatex Research Center, 2014).

[b2] Kaplan K. Quantifying IHC data from whole slide images is paving the way toward personalized medicine. MLO Med Lab Obs. 47, 20–21 (2015). 26742266

[b3] Huang X., Chen S. & Tietz E. I. Immunocytochemical detection of regional protein changes in rat brain sections using computer-assisted image analysis. Journal of Histochemistry & Cytochemistry 44, 981–987 (1996). 8773563 10.1177/44.9.8773563

[b4] Matkowskyj K. A., Cox R., Jensen R. T. & Benya R. V. Quantitative immunohistochemistry by measuring cumulative signal strength accurately measures receptor number. Journal of Histochemistry & Cytochemistry 51, 205–214 (2003). 12533529 10.1177/002215540305100209

[b5] Helps S. C., Thornton E., Kleinig T. J., Manavis J. & Vink R. Automatic nonsubjective estimation of antigen content visualized by immunohistochemistry using color deconvolution. Applied Immunohistochemistry & Molecular Morphology 20, 82–90 (2012). 22157059 10.1097/PAI.0b013e31821fc8cd

[b6] Taylor C. & Levenson R. M. Quantification of immunohistochemistry issues concerning methods, utility and semiquantitative assessment II. Histopathology 49, 411–424 (2006). 16978205 10.1111/j.1365-2559.2006.02513.x

[b7] Ehteshami Bejnordi B. . Stain Specific Standardization of Whole-Slide Histopathological Images. IEEE Trans. Med. Imaging 35, 404–415 (2016). 26353368 10.1109/TMI.2015.2476509

[b8] Macenko M. . A method for normalizing histology slides for quantitative analysis. In 2009 IEEE 6th IEEE Int. Symp. Biomed. Imaging, 1107–1110 (IEEE, 2009).

[b9] Niethammer M., Borland D., Marron J. S., Woosley J. & Thomas N. E. Appearance Normalization of Histology Slides. Mach. Learn. Med. imaging. MLMI (Workshop), author 6357, 58–66 (2010). 10.1007/978-3-642-15948-0_8 PMC4211434 25360444

[b10] Kothari S. . Automatic batch-invariant color segmentation of histological cancer images. In 2011 IEEE Int. Symp. Biomed. Imaging From Nano to Macro, 657–660 (IEEE, 2011). 10.1109/ISBI.2011.5872492 PMC4983436 27532016

[b11] Li X. & Plataniotis K. N. A Complete Color Normalization Approach to Histopathology Images Using Color Cues Computed From Saturation-Weighted Statistics. IEEE Trans. Biomed. Eng. 62, 1862–73 (2015). 25706507 10.1109/TBME.2015.2405791

[b12] Vicory J. . Appearance normalization of histology slides. Comput. Med. Imaging Graph. 43, 89–98 (2015). 25863518 10.1016/j.compmedimag.2015.03.005 PMC4769595

[b13] Alsubaie N., Raza S. E. A. & Rajpoot N. Stain deconvolution of histology images via independent component analysis in the wavelet domain. In 2016 IEEE 13th Int. Symp. Biomed. Imaging, 803–806 (IEEE, Prague, 2016).

[b14] Rabinovich A., Agarwal S., Laris C., Price J. H. & Belongie S. Unsupervised Color Decomposition Of Histologically Stained Tissue Samples. In Adv. Neural Inf. Process. Syst. 16, 667–674 (MIT Press, Vancouver, 2004).

[b15] Khan A. M., Rajpoot N., Treanor D. & Magee D. A nonlinear mapping approach to stain normalization in digital histopathology images using image-specific color deconvolution. IEEE Trans. Biomed. Eng. 61, 1729–38 (2014). 24845283 10.1109/TBME.2014.2303294

[b16] Xu J. . Sparse Non-negative Matrix Factorization (SNMF) based color unmixing for breast histopathological image analysis. Comput. Med. Imaging Graph. 46, 20–29 (2015). 25958195 10.1016/j.compmedimag.2015.04.002

[b17] Ruifrok A. C. & Johnston D. A. Quantification of histochemical staining by color deconvolution. Anal. Quant. Cytol. Histol. 23, 291–9 (2001). 11531144

[b18] Van Eycke Y.-R. . Image normalization for quantitative immunohistochemistry in digital pathology. In 2016 IEEE 13th Int. Symp. Biomed. Imaging, 795–798 (IEEE, Prague, 2016).

[b19] Van Eycke Y.-R. . High-throughput analysis of tissue-based biomarkers in digital pathology. In 2015 37th Annu. Int. Conf. IEEE Eng. Med. Biol. Soc., 7732–7735 (IEEE, 2015). 10.1109/EMBC.2015.7320184 26738084

[b20] Boutsidis C. & Gallopoulos E. SVD based initialization: A head start for nonnegative matrix factorization. Pattern Recognit. 41, 1350–1362 (2008).

[b21] Decaestecker C. . Requirements for the valid quantification of immunostains on tissue microarray materials using image analysis. Proteomics 9, 4478–94 (2009). 19670370 10.1002/pmic.200800936

[b22] Onder D., Zengin S. & Sarioglu S. A Review on Color Normalization and Color Deconvolution Methods in Histopathology. Appl. Immunohistochem. Mol. Morphol. 22, 713–719 (2014). 24897076 10.1097/PAI.0000000000000003

