# Supplementary material for: Image processing in digital pathology: an opportunity to solve inter-batch variability of immunohistochemical staining
Source: Sci Rep. 2017 Feb 21;7:42964. doi: 10.1038/srep42964 (PMC5318955; doi:10.1038/srep42964)
Supplement: Supplementary Information [file srep42964-s1.pdf]

IMAGE PROCESSING IN DIGITAL PATHOLOGY: AN OPPORTUNITY TO SOLVE INTER-BATCH VARIABILITY OF IMMUNOHISTOCHEMICAL STAINING

Yves-Rémi Van Eycke, Justine Allard, Isabelle Salmon, Olivier Debeir, Christine Decaestecker

Supplementary information on image processing methods and software/ hardware specifications

The cited references are those which are mentioned in the main text.

Blind colour deconvolution methods

Method 1, labelled "Macenko" adapted from[8]. This method aims to identify new axes on the principal plane (defined by the first two principal components) to fit the colour cone. To extract these axes the angle between each pixel and the 1st principal component is computed. For IHC images the projection of the "B" unit vector from the RGB space is taken as a reference to ensure that the "pure" blue pixels correspond to negative angles. To remove the influence of the numerous pixels located near the origin only the angles of the pixels located sufficiently far from the origin (i.e. with a distance > 0.15 in the PC plane) are considered[8]. The new blue and brown axes are respectively defined by the coordinates in the 3D SDA space of the two pixels corresponding to the 1st and 99th percentiles of the angle distribution computed in the PC plane.

Methods 2 and 3, respectively labelled "Li-init" and "Li-init-NMF", adapted from[11]. The initialisation step, "Li-init", is done by converting the RGB space into HSV. A saturation-weighted hue histogram is then computed and K-means clustering is used (with $K = 2$) to find 2 cluster centres that correspond to the hue (H) values of the stain colours. For each H value so extracted from the histogram, corresponding S and V values are determined to provide HSV vectors by computing a saturation-weighted mean of both the S and V values on a set of pixels close to the cluster centre. The two so extracted HSV vectors are converted back to RGB and then to SDA in the IHC context. To extract the final vectors, an NMF algorithm is then used to decompose non-negative matrix $\mathbf{X}$ into two other non-negative matrices, $\mathbf{M}$ and $\mathbf{C}$ (see equation (S1)). The complete process is labelled "Li-init-NMF".

$$\min ||\mathbf{X} - \mathbf{MC}||_F^2 \text{ with } \mathbf{M} \text{ and } \mathbf{C} \geq 0 \qquad \text{(S1)}$$

Methods 4 and 5, respectively labelled "SNMF" and "Li-init-SNMF", adapted from[16]. SNMF uses an objective function in which regularisation and sparsity terms are introduced (see equation (S2)).

$$\min_{X,C} \frac{1}{2}||\mathbf{X} - \mathbf{MC}||_F^2 + \frac{\alpha_1}{2}||\mathbf{M}||_F^2 + \frac{\alpha_2}{2}||\mathbf{C}||_F^2 + \frac{\beta}{2}\sum_{i=1}^{n}||\mathbf{C}(:,i)||_1^2 \text{ with } \mathbf{M} \text{ and } \mathbf{C} \geq 0 \qquad \text{(S2)}$$

where α_1, α_2 and β are positive constants. The two terms in the middle of the equation penalise large values in $\mathbf{M}$ and $\mathbf{C}$. The last term is the sparsity constraint.

Xu et al.[16] propose to use 2.5 as a common value for α_1, α_2 and β on the basis of an experimental and qualitative analysis. In our application to IHC images setting this constant resulted in null stain vectors with the two initialisation techniques mentioned in the main text (cf. subsection "Blind colour deconvolution"). After experimental tests we set the 3 parameters to 0.2 and tested this method with the two initialisation techniques, providing us two variants labelled "SNMF" and "Li-init-SNMF".

SDA distribution fitting

We tested two methods proposed in the literature[8,15]. In the first method[8] deconvoluted OD distributions are fitted by simply multiplying each value by the ratio of the 99th percentiles ($P99$), i.e. $P99_{t,c}/P99_{k,c}$, where t is the target image, k is the image to normalise with regard to t and c is the deconvoluted channel. Khan et al.[15] use B-Splines to fit intensity distributions (without OD transform) for each deconvoluted channel. The splines are fitted on 5 values, including 3 distribution parameters (5th percentile, mean and 95th percentile) and the theoretical minimum and maximum values (e.g. 0 and 255 in intensity values) to impose the identity at these extreme values. To apply this method on SDA directly we set the identity at 0 and $-\log 1/255$. In our application to IHC images we use cubic smoothing splines to implement this method. We also generalise these two methods by either applying linear regression or B-spline regression on quantile-quantile (Q-Q) plots constituted of regularly distributed quantiles sampled from the $SDA_{dec,k,c}$ value distributions. We use the 100 percentiles for fitting linear regression whereas we fit cubic smoothing splines on 20, 30 and 100 quantiles regularly spaced and always beginning at the 1st percentile.

Software and hardware specifications

NDPI images were generated using NDP.Scan 2.5, the proprietary software of Hamamatsu. Tiles were extracted using NDP.Read 1.1 (Hamamatsu) and were written to the PNG format using Python 2.7 along with scikit-image 0.11 or in the proprietary NDPI format using NDP.Write 1.1.2 (Hamamatsu). Like previously done for the method developed to identify each core in a TMA slide image[19], the code of the proposed methodology was written in Python using numpy 1.10, scipy 0.17, scikit-learn 0.17, nimfa 1.2. The machine was a laptop running a 64-bits version of windows 10 with a Core I7-6700hq CPU, 16GB of ram and an SSD (solid-state drive) with a minimal overhead. In these conditions the computation time usually took 20 minutes to process a batch of 8 whole slides (scanned at 20x). The code could be optimised by rewriting it in another language or using the GPU. The overhead was totally manageable in our IHC image analysis workflow.

Supplementary information on immunohistochemistry

Technical, fixative and negative IHC controls were prepared on TMA slides using haematoxylin-eosin, anti-vimentin and (anti-mouse or anti-rabbit) IgG staining, respectively, as previously detailed[21].

Immunostains were performed on Ventana discovery XT (Ventana, Roche Diagnostics, Vilvoorde, Belgium) using a Discovery DABMap Detection Kit (P53, PDGFRA and IGF2R) or a Discovery ChromoMap DAB Kit (ERG), both from Ventana. Briefly, deparaffinized and rehydrated (5µm-thick) tissue sections were incubated in EDTA buffer (pH 8.4) and endogenous peroxidase activity was inhibited using Endogenous Biotin Blocking Kit. After primary antibody incubation the slides were washed and incubated with a secondary antibody against mouse (for P53) or rabbit (for the other targets). The HRP binding secondary antibody was detected by incubation with diaminobenzidine (DAB) and hydrogen peroxide. Sections were counterstained with haematoxylin (HEM) and mounted. Additional details on the experimental conditions can be found in Table **S1.** Except for the CD3 experiment, the different IHC batches were carried out with the same lots for the antibodies and the kits.

Antibody	Provider	Dilution	Incubation time	Incubation temperature
**Anti-ERG (Rabbit monoclonal, EPR3864)**	Roche (790-4576)	Ready to use	12 minutes	37°c
**Anti-IGFIIR (Rabbit polyclonal)**	Santa Cruz (sc-25462)	1 :50	60 minutes	37°c
**Anti-P53 (Mouse monoclonal, Bp53-11)**	Roche (760-2542)	Ready to use	40 minutes	37°c
**Anti-PDGFRa (Rabbit polyclonal)**	Thermoscientific (PA5-16742)	1 :100	4 hours	Room temperature
**Anti-CD3 (Rabbit monoclonal, 2GV6)**	Roche (ref : 790-4341)	Ready to use	16min	37°c
**Mouse IgG1 isotype control (monoclonal)**	DAKO, X0931	Conditions used with the associated primary antibody		
**Rabbit IgG isotype control (polyclonal)**	Abcam, ab27478	Conditions used with the associated primary antibody		

Table S1: Details on the IHC assays

Supplementary Figures

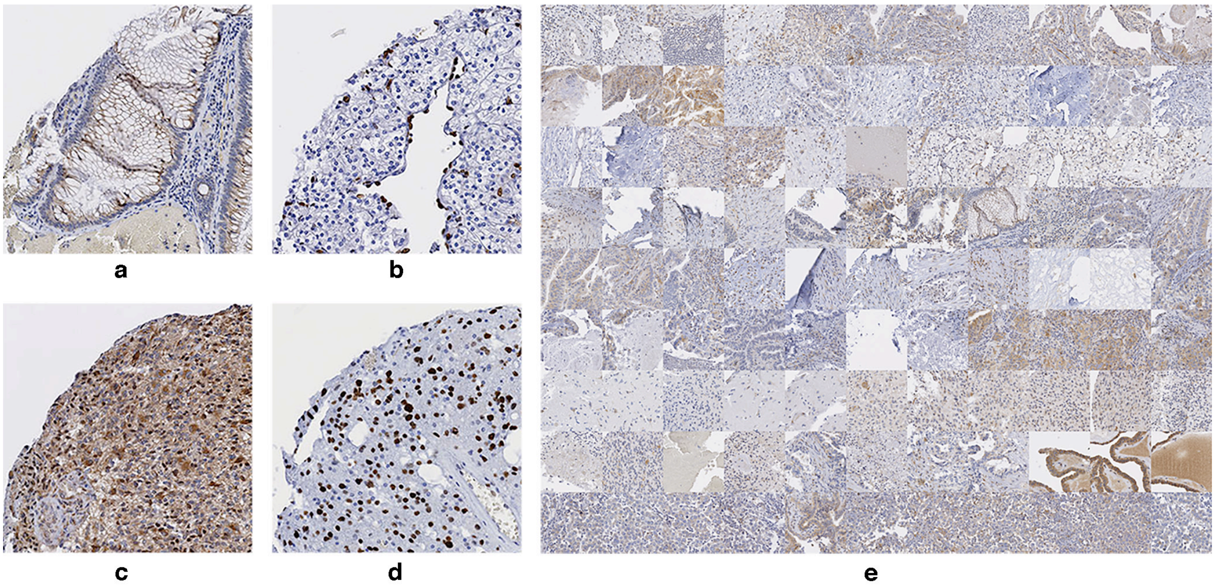

Figure S1: Examples of IHC staining using DAB and HEM on a TMA core (partially shown): a) IGF2R in colon, b) ERG in liver, c) PDGFRa in glioblastoma, d) P53 in glioblastoma. e) Tiled image characterising an IHC experiment targeting IGF2R and extracted from a TMA slide composed of tissue samples from various origins.

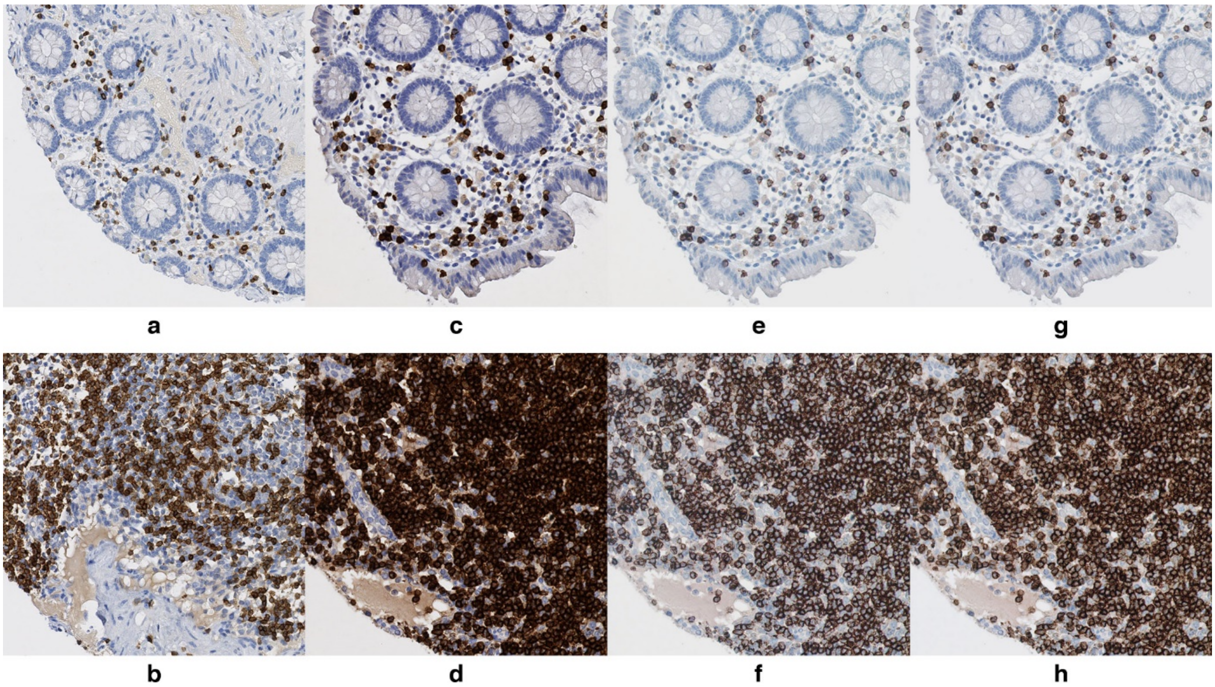

Figure S2: Application of the normalisation methodology, using "Macenko" for colour matching and a regression with 100 splines for SDA fitting, to reduce the variations between the CD3 batches. Illustration of CD3 expression evidenced in cores from a-b) the batch of reference, c-d) the batch to normalise and the corresponding images obtained after normalisation (colour and SDA fitting) based on a selection of 21 cores (e-f) or 43 cores (g-h).

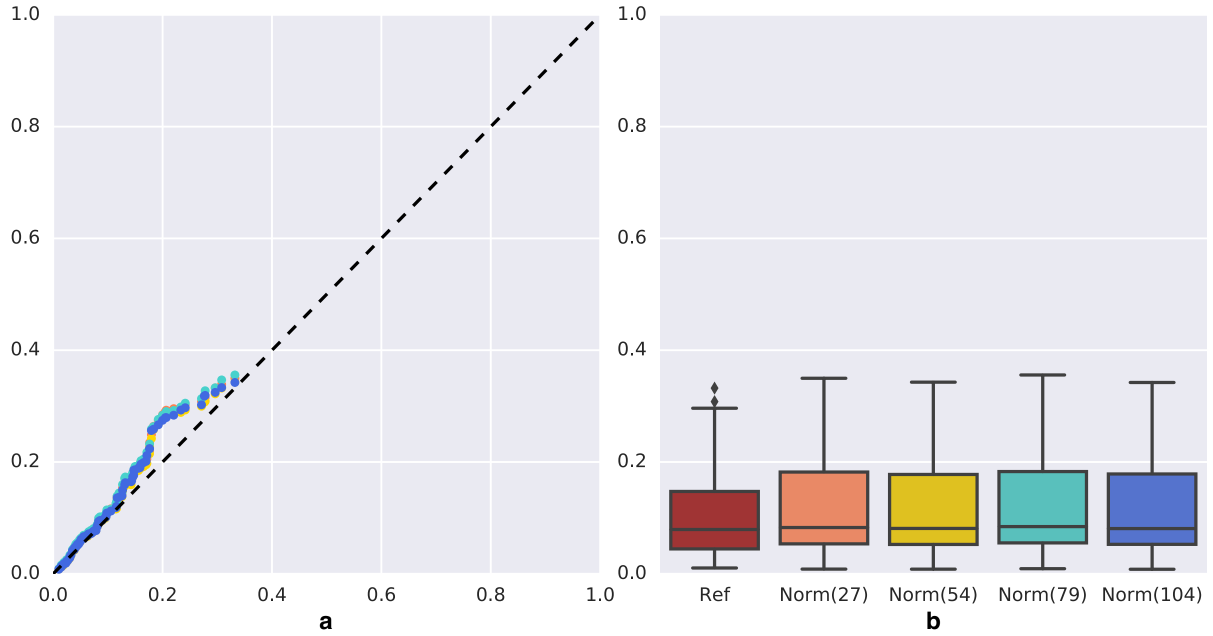

Figure S3: Impact of the number of cores used for image normalisation for the ERG experiment. a) Q-Q plots (X: reference batch, Y: batch to normalise) comparing the ERG LI distributions obtained after batch 2 normalisation (using "Macenko") based on 27 (orange), 54 (yellow), 79 (turquoise) and all (blue) of the available TMA cores. b) Box plot of the ERG LI computed for batch 1 (ref) and normalised batch 2 in the different conditions. Box boundaries = 1st and 3rd quartiles, inner horizontal line = median, whiskers = minimum and maximum values.

Supplementary table (related to the results)

**CD3**<br>***(n=43)***	**LI**<br>**KS statistic**	**LI**<br>**KS p-value**	**QS**<br>**KS statistic**	**QS**<br>**KS p-value**
**Before colour matching**	0.29	0.05	0.33	0.01
**After colour matching (Macenko)**	0.26	0.09	0.31	0.03
**After additional SDA fitting (B-splines)**	0.12	0.91	0.14	0.75

Table S2: Comparison of the LI and QS distributions obtained for CD3 before and after each normalisation step.
